# Coping behavior toward occupational health risks among construction workers: determinant identification using the COM-B model and data mining analysis

**DOI:** 10.3389/fpubh.2025.1643332

**Published:** 2025-09-12

**Authors:** Xuesong Yang, Yuyan Ling, Liqun Wang, Yiqi Li, Mingrong Zeng

**Affiliations:** ^1^Institute of Safety Production Theory and Standards, China Academy of Safety Sciences and Technology, Beijing, China; ^2^School of Emergency Management and Safety Engineering, China University of Mining and Technology-Beijing, Beijing, China

**Keywords:** behavior change technique, COM-B model, association rule analysis, coping behavior, occupational health risk, construction worker

## Abstract

**Background:**

China has the largest construction workforce in the world but faces severe occupational health challenges. Coping behaviors related to occupational health risks (CBOHR) are key to mitigating these hazards but remain understudied.

**Materials and methods:**

A cross-sectional survey of 484 construction workers was conducted to assess Capability, Opportunity, Motivation, and Behavior using the COM-B model. Structural equation modeling (SEM) was employed to test mediating pathways, and association-rule mining (ARM) was used to identify determinants of high- and low-level CBOHR.

**Results:**

The results showed that the COM-B framework—comprising three modules (Capability, Opportunity, and Motivation) with 15 behavior change domains, and a Behavior module with eight specific CBOHRs—demonstrated satisfactory fit, reliability, and validity. Bootstrapping confirmed that Motivation fully mediates the relationship between Capability and Behavior and partially mediates the relationship between Opportunity and Behavior. ARM further identified key domains associated with high and low levels of CBOHR.

**Conclusion:**

Strongly correlated item sets identified through association rule analysis revealed domains strongly linked to both high (and low) levels of each CBOHR. This study is the first to integrate the COM-B model with data mining in the context of occupational health, highlighting “motivation–values–policy” as actionable levers for CBOHR interventions. The findings provide preliminary evidence to support the development of scalable worker health programs.

## 1 Introduction

Work-related health risks faced by workers in the construction industry stem from a wide range of occupational hazards, such as noise, dust, heat, musculoskeletal disorders (MSDs), and work stress (1–4). The large scale of the construction industry presents many obstacles to the prevention of health damage in this sector, especially in developing economies such as China, where rapid urbanization and infrastructure development have led to a huge number of construction projects (5). However, currently, the temporary nature of construction projects and the large number of migrant workers make it inevitable that immediate safety concerns take precedence over long-term health concerns, due to cost constraints and limited access to medical services (6).

Unlike the control of unsafe behaviors, where managers' accomplishments can be assessed based on certain indicators, such as the frequency of accidents and incidents (5, 7). Health-risking behaviors occur often in the workplace and may go unnoticed by both employees and supervisors. There are no explicit documents to police or monitor workers' self-protective health measures in China's construction sector. For instance, OHS supervisors are unlikely to notice if a worker does not wear a mask or fails to take precautions to protect physical health at work. On the other hand, a worker who does not wear a helmet at work may receive a warning or even a penalty (8). That is, chronic health problems do not lead to disputes over rights and responsibilities between the organization and the individual and may not affect the direct interests of the company. Despite everyone agreeing to further protect workers' health, few companies take this step, especially for frontline workers.

Various factors contribute to work-related ill health, such as unavoidable occupational hazards, organizational management issues, government regulations, and personal lifestyles, in which individual self-management of occupational health is easily overlooked. The competence of workers to adopt efficient risk-coping behaviors when confronted with occupational health hazards can be interpreted as worker health self-management. Notably, when construction workers are faced with occupational health risk challenges, they often do not choose to take proactive action in coping (9, 10). Coping behaviors related to occupational health risk (CBOHR) are complex, with changing risk scenarios, yet integrated behavior change theories have often been overlooked in behavior change efforts. For interventions of risk-coping behavior change, construction workers remain an understudied occupational group who address health damage from occupational health hazards through self-response.

There is currently a knowledge gap in how more complex factors of behavior change determine effective coping when construction workers are faced with occupational health risk situations. The Theoretical Domains Framework (TDF) was considered to systematically determine barriers and facilitators of CBOHR. It explained the factors on health-related behavior change (11, 12). In addition, for data mining of the behavior change model, association rules appear to be a useful tool for problem-solving. Data mining approaches using association rules have been utilized to identify crucial influencing elements by discovering valuable associations concerning the outcome variables (13, 14). This technique can assist researchers in more precisely identifying the critical elements that lead to high-level or low-level coping behaviors. This enables a deeper understanding of the factors that facilitate or impede coping behavior change. To address the dilemma of occupational health management for construction workers, classical statistical modeling coupled with association rule data mining may offer a guide for worker health behavior interventions.

The goals of this study are to address the present gap in CBOHR among construction workers. (1) A literature review was conducted to identify potential determinants of CBOHR for construction workers from domains connected to behavior change and to formulate hypotheses. (2) Measurement tools for CBOHR-related constructs were developed for this study based on the scientific scale development methods. (3) Establishing a model for CBOHR and using quantitative analysis to identify predictors of target behaviors and effect pathways among modules. (4) Based on the association rule approach, strong associations between high-level behaviors and low-level behaviors were extracted to further understand the essential factors of CBOHR. The method this paper employed was coupling the hypothesis-testing” paradigm with the association rule technique to accurately identify determinants and mechanisms of CBOHR, which can help managers and practitioners to develop more scientific intervention strategies.

## 2 Literature review

### 2.1 Coping with occupational health risk

#### 2.1.1 Occupational health risk

Health risk represents the process by which exposure to risk factors leads to disease burden, ultimately enabling the identification of convincing causal relationships between risk–outcome pairs–that is, the derivation of risk factors from their attributable health consequences (15). Occupational health risk assessment and occupational health risk management are the two main components of the scientific field of occupational health risks (16). The former focuses more on quantitative or qualitative calculations of objective health risks from exposure to hazards using deterministic or uncertainty analysis (17–19). Risk management is the adoption of a series of control measures in response to the existence of risks, which may be from organizational and individual behaviors (20, 21). Health-risk factors were divided into five main categories through reviewing published global studies on occupational health management of construction workers, namely, physical hazards, chemical hazards, behavioral hazards, psychological hazards, and biological hazards, which accounted for approximately 51.4%, 19.5%, 15.4%, 12.0%, and 1.7% of research attention, respectively (22). Through epidemiological surveys, construction workers had a significantly higher risk of work-related diseases, especially respiratory, skin, and musculoskeletal diseases, than workers in other industries (23). In addition, the incidence of work-related substance abuse, sleep disorders, psychiatric disorders, and mania had risen significantly for young construction workers (24). Studies on health risks of construction workers are mainly based on epidemiological surveys and psychometric paradigms (i.e., validated questionnaire-based measurement), with epidemiological surveys mainly including physiological functional data by cohort, controlled as well as cross-sectional studies (25–29); questionnaires, scales, and interviews involved in psychometric paradigms are commonly used (10, 30, 31). The last decade has seen the rise of approaches combining subjective psychometric paradigms with intervention trials, simulation experiments, and data mining techniques (14, 32–34). Since health risks are inherently uncertain, future research should focus on improving the accuracy of data collection and analysis, and more fine-grained metrics for risk should be developed to streamline the knowledge structure of occupational health management.

#### 2.1.2 Coping with risks

People evaluate the risk when they become aware of a threat or danger, and they respond by adopting specific activities or procedures to cope with the risk scenario (16, 35). Coping was defined as “changing cognitive and behavioral efforts to manage specific external and/or internal needs” and was guided by two core concepts, approach and avoidance, in the context of stressors, for example, that are oriented either toward or away from threat (36–38). Adopting a response is usually done in a dynamic change of risk perception; however, because the generation of coping tendencies is intra-individual and subjective, there have not been many studies specifically on how construction workers cope with occupational health risks (9). Risk perception is a highly individualized aspect of risk management that frequently serves as a crucial connection to risk management programs. Risk perception is a mental construct, often shaped by cognitive biases, that plays a critical role in shaping responses to risk and serves as a crucial connection to effective risk management programs (39). The field of health risk coping primarily focuses on coping tendencies or competencies related to intra-individual characteristics and external stimuli, with an emphasis on explaining the mechanisms that generate coping responses to health challenges through a psychological paradigm (40–42). The scale of COPE that was developed in 1989 separated 13 conceptually distinct subscales, which were further divided into problem-focused coping and emotion-focused coping (43). COPE is still a useful tool for researching how people respond to health challenges and issues, particularly how the public and medical professionals cope with health risks during the COVID-19 pandemic (44, 45). Coping skills are usually related to personality, experience (or knowledge), and social and environmental influences (46). For construction workers‘ risk-coping competence, occupational safety was given more importance, indicating that research should focus more on coping with occupational health risks. The exploration of the mechanisms of the construction workers' health risk coping model should be facilitated based on conventional coping-related theories and research information in other fields of health risk.

#### 2.1.3 Coping behavior of health risk

The majority of research on construction worker health management focused on risk factors and health intervention strategies from an organizational perspective (47–49). Such studies have regarded these responses as health-protective behaviors. The purpose of health-protective behaviors is to achieve desirable health outcomes. In the studies of health behavior, it was classified as preventive health behaviors (actions taken to avoid illness or injury) and sick-role behaviors (actions taken after diagnosis of a medical problem to restore good health or to prevent further disease progress) (50), or as frequent and infrequent behavior (51). Based on the frequency of behavior implementation, health behaviors are further classified more broadly: frequent preventive behaviors, for example, lifestyle; infrequent preventive behaviors, including disease screening and health assessment; and disease management behaviors, including prevention and control of disease progression (52). Protective motivation (intention) (53), health literacy (54), personality attributes [responsibility (55), optimism (56), and so on], and self-efficacy (57), to varying degrees, play a distinct role in preventative health-protective behaviors when faced with high health risks. In terms of occupational health protective behaviors, they should be considered as preventative health-protective behaviors (both frequent and infrequent prevention) mainly aiming at preventing the incidence of occupation-related ill health and increasing the wellbeing of workers. Further research is necessary to determine, in occupational health settings, the person variables that make behavior occur or change.

The viewpoint of our research is that health-protective behavior is more appropriately called the coping behavior of occupational health risk (CBOHR) in occupational health. This highlights the specific health effects of occupational hazards in the workplace and emphasizes the countermeasures taken to prevent these damages. Many health-protective behaviors are appropriately used for daily health maintenance or health promotion. In the workplace environment of construction workers, however, health damage caused by occupational hazards must be different from that in daily life. Taking measures to cope with the risks brought by occupational hazards and thereby protecting their own health from damage is considered a key component of workers' competence for occupational health self-management.

### 2.2 COM-B model of behavior change

#### 2.2.1 Theoretical domains framework

In order to clarify the theoretical understanding of behavioral change processes, 61 experts from the fields of psychological theory, health services research, and health psychology developed a consensus set of key theoretical constructs (Theoretical Domains Framework, TDF), consisting of 12 domains, which was published in 2005 (12). A group of behavioral scientists who were unaware of the original framework domains then assessed the validity of the domain framework through an empirical base and reordered the 112 unique theoretical constructs, resulting in a 14-domain framework (11). The TDF, which was a framework rather than a theory, was created through expert consensus categorization and compilation of 128 component constructs from 33 theories pertinent to behavior-related implementation issues. Its purpose was to assist users in identifying the critical factors influencing behavior change. Over 800 peer-reviewed articles employing this framework have been published in Web of Knowledge in the past decade since its initial design was made available (58). The description of the domains and theoretical constructs in the TDF is shown in Supplementary Table S1.

The framework was developed to make the many behavior change theories more comprehensible to the interdisciplinary audiences involved in implementation and can be used to understand the barriers and facilitators of behavior change in a range of settings, facilitating the development of behavior change techniques and the design of interventions (59). The initial application of the TDF was to qualitatively and quantitatively analyze the behavior change among healthcare practitioners, which has been widely used in the clinical field. Thus, more research is needed on healthcare practitioner-specific barriers and facilitators of behavior. As TDF research is growing, more researchers are attempting to apply it across a wide range of behavioral interventions, such as occupational safety behavior change (60, 61), physical activity (62), and safety behaviors related to patients (63). Designing questionnaires based on TDF may be a quicker option to pinpoint the key factors of behavior change in a larger sample (64).

#### 2.2.2 COM model

Construction workers are confronted with a multitude of work-related health issues, and their coping behaviors may exhibit overlapping and transformative characteristics. Consequently, health risk-coping behaviors have not received adequate scholarly attention. Furthermore, the lack of authoritative measurement tools and behavioral capture methodologies has posed a series of obstacles to conducting in-depth scientific research on health interventions for construction workers. Behavior change theories can explain coping behavior and interventions based on theories that aim to promote work-related health and wellbeing (40, 65, 66). Behavior change interventions aim to target the prevalence or incidence of particular behaviors in specified populations. A large number of studies rely on specific theories; however, a more integrative cross-theoretical approach should be employed to explore a wider array of constructs as potential behavioral determinants, thereby increasing the likelihood of a successful intervention. The determinations of behavior change were summarized and reorganized in the TDF, but without further theorizing their interactions, leading to the proposal of a new behavior change model framework (termed the “COM system”) containing three key modules: Capability, Opportunity, and Motivation (67). A theoretical model of behavior change was formed by mapping the TDF domains to the COM-B system. The three fundamental conditions of Capability (ability), Motivation, and Opportunity that affect behavior were frequently included in the study of behavioral effects as a global model, derived from the integration of multiple behavioral theories (68–70). The COM-B model has the advantage of mapping the domains in the TDF in its system and serving as the theoretical core of a behavioral intervention pattern that takes into account comprehensiveness, coherence, and a clear link to an overarching behavioral model (67).

After a decade of growth, the COM-B model framework is currently the foundation for an increasing amount of behavior change research, which continues to develop its potential in many fields.

An increasing body of research has explored healthcare practitioner-specific barriers and facilitators of behavior change across a wide range of clinical situations, such as protective behaviors for oral and dental health (71, 72), management of patient health behaviors (73, 74), and medication use or treatment decision-making (75, 76), and often identifying validated variables as key explanatory factors. Additionally, in some practices of public health, researchers are interested in investigating the efficacy of behavior change intervention models. Examples of these areas of practice include specific behavior interventions for children or adolescents (77, 78), physical activity and exercise (79, 80), family health care behaviors (81, 82), control of unhealthy lifestyle behaviors (such as smoking cessation, weight management, and d substance abuse) (83–86), and behavior of environmental protection (87, 88). Numerous studies have applied the COM-B model to workplace health-promoting behaviors. For instance, interventions targeting sedentary behavior among office workers (89, 90) demonstrated that the COM-B model predicted sitting behavior more effectively, albeit marginally, than competing single models in terms of variance explained (91). The COM-B model also appeared beneficial for understanding occupational safety behaviors, such as explaining the low rate of helmet use among farmers operating machinery (92) and ways to improve the appropriate use of personal protection equipment (PPE) by ward staff during the COVID-19 pandemic (93). Many studies used this model to demonstrate that capability and opportunity are associated with target behaviors through the mediating effect of motivation, and they are also directly associated with behavior to some extent.

The COM-B model framework has proven its excellent performance in many fields (non-clinical medicine), and its applications are likely to become more widespread in the future. It has seldom been taken into account in the current field of behavior change of occupational health, and it is necessary to introduce this integrated model for construction workers. The constructs in the COM-B model that facilitate and hinder these behaviors must therefore be used to identify the important factors underlying CBOHR. However, existing COM-B research has yet to disentangle the differential impacts of “capability–motivation” vs. “opportunity–motivation” on low-resource workers.

### 2.3 Association rule mining

Association rule analysis, originally proposed by Agrawal, yielded notable findings from market basket data and has since become a classic example in data mining (94). The primary concept behind this approach was to use an effective algorithm to mine the association rules between sets of items in a large basket of data (items bought by a customer over a period of time in a supermarket) to form the antecedent (X) and infer the consequent (Y), typically expressed as “X → Y.” The most important parameters in the algorithm included Support, Confidence, and Lift, and the results that satisfy these parameters at the same time are considered valuable rules (95).

In the research field of occupational health and safety, association rule mining (ARM) methods were acceptable and provided powerful scientific data to support safety or health improvement. The analysis of the connections between illness absence and lifestyle, as well as medical outcomes among 6,010 employees of a Japanese telecommunication company, was conducted using ARM, and the findings provided valuable guidance for avoiding sickness absence at work (96). The investigation of variables affecting retired athletes' levels of life satisfaction and loneliness by transforming questionnaire data into binary attributes and using a tree-based frequent item set mining method to discover association rules demonstrated greater validity (97). ARM was used to explore the relationship between healthcare workers' level of awareness, preparedness to manage suspected patients, and case-management training received, using multicenter and cross-sectional data in 59 countries during the COVID-19 pandemic, providing proof in favor of increased training opportunities (98). Female homeworkers' health concerns were frequently disregarded, even though they confront major health and safety challenges, while ARM can assist managers to discover factors that impact female homeworkers' exposure to occupational ill health (99). ARM has also become a popular approach in the field of occupational safety research, particularly in the prevention of workplace accidents and incidents. ARM techniques were used to discover cause-and-effect patterns of steel plant accidents from accident investigation data (100) and to explore correlations among human factor events in nuclear power plants, improving a weight association rule based on statistics (101). Cross-sectional data from questionnaires or interviews, as well as injury and incident data recorded, were utilized in research on construction accident prevention to extract novel insight into construction occupational safety using ARM techniques (102–105). There was a clear connection between construction accident prevention and unsafe behaviors, with various unsafe behavior categories frequently being associated with various accident outcomes (106). The mechanisms influencing construction workers' unsafe behaviors were complex and multifactorial, and the correlations among the characteristics of unsafe behaviors had been studied based on ARM (107). ARM through questionnaire data was a new attempt in occupational health and safety in the construction industry, which seems to explain cause-and-effect patterns well (14).

### 2.4 Research hypothesis

In this study, we initially conducted the design and testing of the Capability, Opportunity, Motivation, and Behavior (target behaviors) as subscales separately. Next, these four modules were integrated into the COM-B model for the quantitative analysis of the roles of Capability, Motivation, and Opportunity in influencing construction workers' risk-coping behaviors.

Although the TDF/COM-B framework has been validated in the fields of healthcare and lifestyle, its applicability in the occupational health context of construction workers still faces two major gaps: (i) high onsite mobility and cultural heterogeneity, which may undermine the effect of social norms; and (ii) cross-sectional evidence that focuses solely on safety behaviors, without examining the dynamic mechanisms of health risk management. Therefore, this study proposes hypotheses H1–H5 to validate the convergent and predictive validity of the COM-B framework in this context.

Hypothesis 1 (H1). Capability is positively and directly associated with coping behavior.

Hypothesis 2 (H2). Opportunity is positively and directly associated with coping behavior.

Hypothesis 3 (H3). Motivation is positively and directly associated with coping behavior.

Hypothesis 4 (H4). Capability is positively indirectly associated with coping behavior through motivation as a mediator.

Hypothesis 5 (H5). Opportunity is positively and indirectly associated with coping behavior through motivation as a mediator.

## 3 Materials and methods

### 3.1 Design of the scale

#### 3.1.1 Generation of items

The items in the Capability and Motivation were referenced to the dimensions mapping under the theoretical domain framework of behavior change to the COM-B model. This is an exploratory study about occupational health-related behaviors of construction workers attempting to make a clear behavior change mechanism through the COM-B model. Morgan introduced the TDF to the measurement of occupational safety behaviors and developed a safety behavior change questionnaire that was valuable to our study (60). Safety and health behaviors at work share overlapping and similar characteristics, linked together through occupational risk (108). Therefore, we adopted Morgan's process for obtaining research evidence on safety behavior change mechanisms based on the TDF. Through publications based on TDF-related questionnaires or interviews, on-site surveys of projects under construction by our research team, and the status of occupational health management of construction workers, we designed the initial items within domains in TDF. And then they were mapped to modules, respectively (Supplementary Table S2).

##### 3.1.1.1 Capability

**Figure 1:**
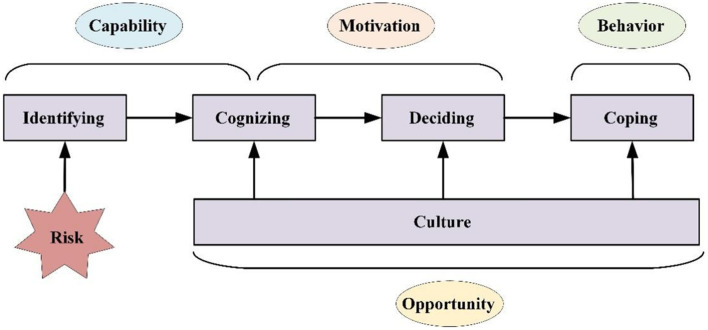
Flowchart for developing the COM-B-based scale.

In the “COM-B” system, Capability was seen as an essential factor of behavior generation. It was defined as “an individual's psychological and physical capacity to engage in the activity concerned”. For occupational health behavior of construction workers, capacity encompasses knowledge, skills, and behavioral habits. Physical capacity is a requirement for a construction worker to be able to get the job, so there is not much physical capacity to consider in occupational health behaviors. We merged knowledge and skills into one construct because construction workers' knowledge and skills regarding occupational health protection are not easily distinguishable. Therefore, under the Capability module, we divided into three latent variables as domains, consisting of four constructs of TDF: “behavioral regulation,” “memory, attention and decision processes,” and “knowledge and skills.” Under each domain, we designed four related preliminary items. The flowchart of subscale development of Capability is shown in Figure 1. Figure 1 delineates a seven-step, empirically validated procedure for constructing the COM-B-based scale, representing a mature scale-development workflow.

##### 3.1.1.2 Motivation

Motivation was considered to be composed of habitual processes, emotional responding, and analytical decision-making, which mainly reflect the direct energy for behavior at the level of individual thought. Motivation has been mentioned in many behavioral theories, and the COM-B model incorporates constructs from relevant behavior change theories. We used seven domains as latent variables, i.e., “emotion,” “social/professional role and identity,” “beliefs about capabilities,” “beliefs about consequences,” “intentions,” “reinforcement,” and “optimism.” The domain called Goals was not well-suited for construction workers' health behaviors in occupational activities, because there are few specific goals for health promotion. Four initial items were designed for each domain. The flowchart of subscale development of Motivation is shown in Figure 1.

##### 3.1.1.3 Opportunity

**Figure 2:**
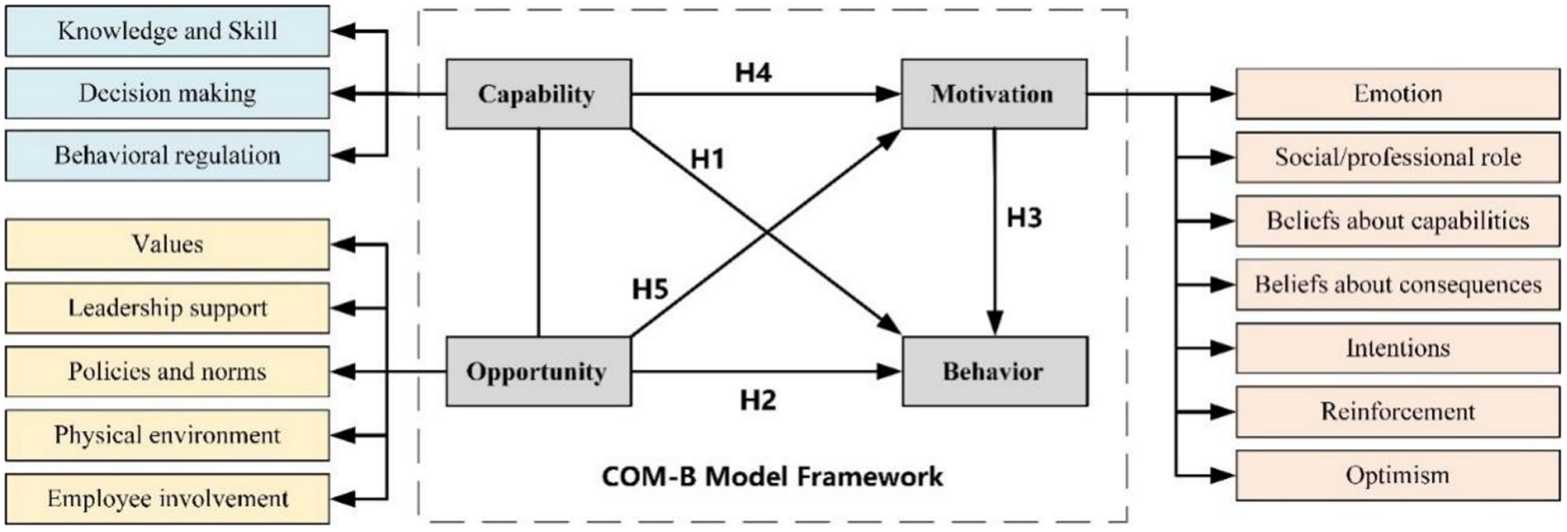
Integrated COM model framework.

Unlike Capability and Motivation, which were intrinsic drivers of behavior change, Opportunity acted as an external force for the individual to influence behavior. Opportunity included physical and social factors afforded by environment and culture, respectively, and was defined as “factors that lie outside the individual that make the behavior possible or prompt it”. In the COM-B model, the module of Opportunity was composed of two domains, “Environmental context and resources” and “Social influence”. We embedded the occupational health culture scale (OHCS) that had already completed in a published study into Opportunity (this scale contains environmental components), including the five domains of “values” (3 items), “leadership support” (5 items), “policies and norms” (5 items), “physical environment” (4 items), and “employee involvement” (4 items) (109). Culture exists as an external factor, but it has to be reflected through the subjective psychological feelings of individuals so that the authenticity and accuracy of the measurement can be ensured. Therefore, we employed OHCS instead of the Opportunity subscale to complete the measurement (the integrated theoretical COM model framework shown as Figure 2).

##### 3.1.1.4 Target behavior

Target behavior was regarded as the dependent variable in this study. The module of behavior was not developed as a subscale because there was no consensus research on the domain delineation of health behaviors among construction workers. Experienced measurement methods used to assess risk-coping behaviors in occupational health for construction workers have rarely been explored. Therefore, many studies have exemplified protective behaviors in occupational health that are unique to construction workers or frequently engaged during their operations. We mainly cited the risk coping behaviors of occupational health for construction workers, summarized by Liu et al. (9). To more scientifically measure the level of construction workers' behaviors, we synthesized eight specific behaviors based on publications, on-site research, and interviews with experienced construction workers. Identifying the target behaviors was a key step in the TDF approach and the outcome variable studied in the COM-B model, which provided a basis for exploring the determinants that facilitate and impede the occurrence of the behaviors. After forming these target behaviors, we conducted a stakeholder consultation, which was similar to some of the methods used to develop questionnaires based on TDF. In order to support the improvement of these items of behavior, comments of experts were adopted (60, 110). The types of eight target behaviors are as follows: medical insurance (Behavior 1, BEH-1), usage of protective equipment (Behavior 2, BEH-2), maintaining ventilation (Behavior 3, BEH-3), physical examination (Behavior 4, BEH-4), avoiding rest and eating in the workplace (Behavior 5, BEH-5), good interpersonal/social relationship (Behavior 6, BEH-6), avoiding musculoskeletal disorders (Behavior 7, BEH-7), and avoiding cold-related or heat-related illness (Behavior 8, BEH-8) (9).

#### 3.1.2 Optimization of items

We have established a fifteen-member group of experts in the fields of health promotion, occupational disease prevention, behavioral science, OHS management, and scale development, including researchers from scientific institutions, OHS managers, industrial hygiene engineers, and occupational health physicians. We convened a panel of experts to refine the scale. For example, frontline field experts believed that the concept of “resources available on the site” might be unclear to construction workers. Therefore, the questionnaire should rephrase the description of these resources to ensure clarity. Formal written language was used in the publication; however, in the questionnaire, some colloquial adaptations could be made appropriately to align with the Chinese language habits while ensuring that the meaning remains intact. As a result, replacing the phrase “I can use some equipment or resources to cope with occupational health risks” with “I can cope with occupational health risks in some ways” would help workers make more accurate choices.

Moreover, at the pre-testing phase, we used the cognitive interview method, which was also adopted and recommended in questionnaire development (111, 112). Fifteen construction workers with over 100 years of experience on construction sites were selected to discuss the design of the items with our researchers. A report of our design and measurement purpose was given to them, allowing them to assess the content validity and reliability of the questionnaire. We obtained the interviewees' feelings about answering the questions and their suggestions for the items.

### 3.2 Sample

In 2021, our research team launched a project called “Standardization of Occupational Health Management in Construction Projects“ with a regional company of a large real estate group in China. At two of the company's construction projects (large residential projects) in southwestern China in May 2021, researchers of this study administered the COM-B questionnaire to a random sample of 450 workers, receiving 409 valid questionnaires. In September 2021, questionnaires were distributed to 79 female construction employees, and 75 valid responses were collected. Participants were included based on the following criteria: able to understand and complete the questionnaire; aged between 18 and 70 years; in good physical condition; and with no history of occupational disease. All workers signed the consent form and completed the study instruments in writing. There were no ethical issues in this survey, and each respondent signed a privacy agreement (the demographic results were presented in Supplementary Table S3).

### 3.3 Data analysis

#### 3.3.1 Statistical analysis

Two subscales, Capability and Motivation, were used for exploratory and confirmatory factor analysis, respectively. We divided the sample (n=484) into two parts, with the first half (sample 1) used for exploratory factor analysis (EFA) and the second half (sample 2) used for confirmatory factor analysis (CFA). Before conducting exploratory factor analysis, we analyzed the sample for inter-item and total item correlations, requiring that the total item correlation for each item not be < 0.5.

In the exploratory factor analysis, this study used principal component factor analysis with the varimax rotation eigenvalue criterion > 1.0 to detect the latent variables. Before identifying the latent variables, we verified the feasibility of factor analysis by using the Kaiser-Meyer-Olkin (KMO) test and Bartlett's sphericity test. Typically, factor analysis is more suitable when the KMO value exceeds 0.8 and the Bartlett's sphericity test *p* < 0.01. And then, items are determined to be retained or removed through factor loadings and cross-loading. In general, the factor loadings should be >0.5 and only in one of the domains (items with cross-loadings >0.5 should be eliminated or modified).

After completing the exploratory factor analysis, we validated the fit results for the domain (factor structure). The confirmation factor analysis of this paper was similar to Morgan's approach, which developed a safety behavior change questionnaire based on the TDF, presenting the CFA in more detail in their study (60). In this process, the exclusion of unsuitable items was made by the modification index (M.I.). The absolute fit index contains chi-squared/degree of freedom (χ2/df), goodness-of-fit index (GFI), adjusted for the model's degrees of freedom (AGFI), root mean square error of approximation (RMSEA), and standardized root mean square residual (SRMR). The relative fit index contains the normal fit index (NFI), the Tucker-Lewis Index (TLI), and the comparative fit index (CFI). Both IBM SPSS 24.0 and IBM AMOS 25.0 were used to complete the exploratory factor and validation factor analysis. In addition, the convergent validity was expressed by the average variance extracted (AVE), and an AVE >0.5 indicates acceptable convergence. The Cronbach's alpha coefficient is usually used to characterize internal consistency of the scale items, with a value between 0 and 1, and the larger the value, the higher the reliability, with 0.70 being an acceptable threshold. The composite reliability (CR) value >0.7 represents an appropriate construct reliability.

#### 3.3.2 Model analysis

The next process was to construct the COM-B overall model and analyze the fit by structural equation modeling (SEM) based on a hypothetical model framework. According to the previous hypothesis, Capability, Opportunity, and Motivation have an effect on CBOHR, and Motivation, meanwhile, acts as a mediator. We performed a structural equation modeling analysis using the maximum likelihood estimation in IBM AMOS 25.0 to observe the influence of the components on the target behavior. Then, the mediating effects analysis was conducted using a bootstrapping approach in Amos software. Indirect effects were assessed using *n* = 5,000 bootstrap re-samples. They were tested for significance after correcting for 95% bias, and the confidence interval (bias-corrected 95% CI and percentile 95% CI) did not include zero.

#### 3.3.3 Association rule analysis

##### 3.3.3.1 Data preprocessing

To investigate an association between diverse CBOHR and domains of behavior changes, the Association Rule Mining (ARM) technique was used to explore the co-occurrence relationship. Data preprocessing was performed to transform the data into a scale and descriptive statistics suitable for ARM. We transformed the quantitative scores of the scales into “High (H),” “Middle (M),” and “Low (L)” qualitative categorizations, respectively. Tercile splits were not considered in this work because, according to our sample normality tests (kurtosis and skewness), the mean scores are clustered around 3, which could result in a very small sample being included in the association analysis (113). Therefore, domains were classified into three degrees: average score < 3 As “low,” 3 ≤ average score < 4 as “medium,” and average score ≥ 4 as “high.” According to the five-point Likert scale we employed, scores of 1 and 2 were negative responses. Although a score of 3 was a positive response, it was usually considered to be close to a neutral response and did not accurately capture the mental attitude of the respondent. Scores of 4 and 5 can be considered as positive responses with a strong attitude.

##### 3.3.3.2 Parameter and rule setting

The significance of association rules is to discover useful rules above the threshold of a set parameter. Setting reasonable parameters is essential for discovering valuable rules. The Apriori algorithm is an optimized classical algorithm that has been widely used and aims to construct frequent item sets using an iterative method on horizontal search. It was found that a minimum support of 5% has high data mining efficiency (94). Similar findings were obtained in the study of Li et al. (14), and their work transformed the questionnaire data into qualitative categories labeled “high” and “low” as well. The minimum parameters were set according to the research expectation and the scale of the data. When the rules satisfy the requirements of minimum Support, Confidence, and Lift (shown in Equations 1–3) at the same time, the strong rules are considered available. Strong rules will be filtered out, and occurrence patterns within strong rules will be analyzed.


(1) $$\text{Support}\left(\text{X},\text{Y}\right)\text{=}\frac{\text{P}(\text{X},\text{Y})\;}{\text{P}(\text{All})}$$

(2) $$\text{Confidence}\left(\text{X}\to \text{Y}\right)\text{= }\frac{\text{P}\left(\text{X},\text{Y}\right)}{\text{P}\left(\text{X}\right)}$$

(3) $$\text{Lift}\left(\text{X}\to \text{Y}\right)\text{= }\frac{\text{P}\left(\text{X},\text{Y}\right)}{\text{P}\left(\text{X}\right)\text{P}\left(\text{Y}\right)}\text{=}\frac{\text{P}(\text{Y}|\text{X})}{\text{P}(\text{Y})}$$

## 4 Results

### 4.1 Factor analysis for subscales

#### 4.1.1 Subscale of capability

In the subscale of Capability, the first exploratory factor analysis yielded four latent variables with eigenvalues greater than one, accounting for 73.18% of the total variance. The KMO test for sampling adequacy was 0.886, and the Bartlett test for sphericity was highly significant (*p* < 0.001). By observing the inter-item and item-total correlations of 12 items, we eliminated items with correlation coefficients < 0.5. Two items (KS3 and DM1) were eliminated because their modified item-total correlations were 0.199 and 0.105, respectively.

Following varimax rotation, the component matrix of the factor structure revealed that KS-3 and DM-1 loaded onto a newly extracted factor. Within this factor, KS-3 had a factor loading of −0.534 and demonstrated a cross-loading of >0.5 (specifically, 0.607) with the “Behavioral regulation” dimension. In contrast, DM-1 had a strong factor loading of 0.817; however, its content showed no substantive conceptual alignment with KS-3. This suggests that DM-1 does not satisfy the theoretical or conceptual criteria necessary to be grouped with KS-3 under the same latent construct, as shown in Supplementary Table S4.

Confirmatory factor analysis was employed to assess the degree of fit between the observed data and the conceptual model. We modified the three-factor model according to the modification index (M.I.) values. BR3 had the highest M.I., and considering that each domain maintained the same number of items, BR3 was removed. The results indicated a good model fit for the latent variable of Capabilities. Model fit indices before and after modifications are shown in Supplementary Table S5.

For modified models, the Cronbach alpha coefficient and CR of each factor exceeded the threshold of 0.70, and the corrected item-to-total correlations were all >0.5, meaning good internal consistency reliability (Table 1). The square root of AVE exceeds the domain correlation, and therefore, the domains presented convergent and discriminant validity, as shown in Table 2.

**Table 1:** Factor loading results for the subscale of capability.

**Domains**	**Items**	**Corrected item-to-total correlation**	**Cronbach's α**	**Factor loadings**			**CR**	**AVE**	**Total explained variance (%)**
				**1**	**2**	**3**			
Behavioral regulation	BR1	0.598	0.867	0.821			0.830	0.621	26.187
	BR2	0.625		0.802					
	BR4	0.799		0.738					
Decision making	DM2	0.607	0.853		0.833		0.833	0.625	25.950
	DM4	0.66			0.812				
	DM3	0.835			0.722				
Knowledge and skills	KS4	0.595	0.842			0.847	0.839	0.636	25.731
	KS2	0.595				0.835			
	KS1	0.798				0.703			

**Table 2:** Correlations between factor structures for the subscale of capability.

**Domain**	**BR**	**DM**	**KS**
BR	—		
DM	0.771^***^	—	
KS	0.760^***^	0.785^***^	—
Square root of AVE	0.788	0.790	0.798

^***^P < 0.001.

#### 4.1.2 Subscale of motivation

There were nine latent variables with eigenvalues greater than one in the exploration factor analysis, and their total variance was 70.98%. The KMO test for sampling adequacy was 0.868, and the Bartlett test for sphericity was highly significant (*p* < 0.001). Item-total, factor loadings, and cross-loadings were likewise used to determine whether the existence of items was appropriate to the scale. In this process, seven items were removed, which were from seven domains. Factor loadings of OP2 and EM3 were < 0.5. INT3 and BCA2 formed a new latent variable, whereas these two items had poor relevance in regard to item content. BCO3 and SR4 formed an uncorrelated latent variable, with a factor of < 0.5 for BCO3; therefore, this latent variable was excluded. Given that a latent variable should involve no less than three items, and the factor loadings are < 0.5 for BCO3, a latent variable formed by BCO3 and SR4 was excluded. While REI3 had acceptable loading factors, the item-total correlation was 0.306, which was also a judgment for item elimination.

The next work confirmed the seven-factor model of Motivation using AMOS 25.0 and put the calculation in Supplementary Table S5. We did not make any modifications to this model because its fit was adequate. Reliability tests on the subscale of Motivation obtained internal consistency reliability, as shown in Table 3. The results of convergent and discriminant validity were calculated as shown in Table 4. These results demonstrated that the subscales of Motivation have reasonable reliability and validity.

**Table 3:** Factor loading results for the subscale of motivation.

**Domains**	**Items**	**Corrected item-to-total correlation**	**Cronbach's α**	**Factor loadings**							**CR**	**AVE**	**Total explained variance (%)**
				**1**	**2**	**3**	**4**	**5**	**6**	**7**			
Social/professional role	SR3	0.59	0.881	0.830							0.867	0.686	11.651
	SR1	0.582		0.827									
	SR2	0.587		0.827									
Optimism	OP1	0.565	0.879		0.859						0.870	0.690	11.603
	OP2	0.578			0.817								
	OP3	0.59			0.816								
Beliefs about consequences	BCO1	0.604	0.877			0.823					0.856	0.670	11.476
	BCO3	0.588				0.816							
	BCO2	0.6				0.816							
Reinforcement	REI3	0.588	0.878				0.830				0.857	0.667	11.416
	REI1	0.604					0.815						
	REI2	0.614					0.804						
Emotion	EM3	0.616	0.871					0.819			0.854	0.661	11.388
	EM2	0.568						0.819					
	EM1	0.612						0.801					
Intentions	INT1	0.579	0.868						0.832		0.861	0.674	11.364
	INT2	0.571							0.824				
	INT3	0.567							0.807				
Beliefs about capabilities	BCA1	0.561	0.851							0.828	0.850	0.654	11.195
	BCA2	0.556								0.802			
	BCA3	0.579								0.796			

**Table 4:** Correlations between factor structures for the subscale of motivation.

**Domain**	**SR**	**OP**	**BCO**	**REI**	**EM**	**INT**	**BCA**
SR	—						
OP	0.459^***^	—					
BCO	0.534^***^	0.447^***^	—				
REI	0.449^***^	0.504^***^	0.535^***^	—			
EM	0.504^***^	0.461^***^	0.507^***^	0.512^***^	—		
INT	0.453^***^	0.446^***^	0.461^***^	0.477^***^	0.484^***^	—	
BCA	0442^***^	0.631^***^	0.470^***^	0.494^***^	0.501^***^	0.519^***^	—
Square root of AVE	0.828	0.831	0.818	0.816	0.813	0.821	0.809

^***^P < 0.001.

### 4.2 Confirmation factor analysis of the model

Through hierarchical confirmation factor analysis and items removed by statistically unreliable indicators, Capability and Motivation as second-order factor models (latent variables) had been shown to have good reliability and validity. We had developed the subscale of Opportunity in the previous study, so this module was only tested with a factor model, and the reasonable fit results are shown in Supplementary Table S1. The results of the global COM-B model indicated that the fit was acceptable: χ^2^/df = 1.196, GFI = 0.885, AGFI = 0.874, NFI = 0.908, TLI = 0.983, CFI = 0.949, RMSEA = 0.00, and SRMR = 0.035. The results of the factor loadings, AVE, and CR calculations for the second-order latent variables are shown in Table 5. Only the second-order latent variables of the Motivation subscale had an AVE value close to the acceptable threshold (AVE = 0.482), while all other second-order latent variables exhibited AVE values within a broader acceptable range. It might be because Motivation encompasses a relatively large range of constructs containing seven domains. Moreover, the influence of Motivation was very complex, and it is reasonable that there are some biases in respondents' understanding and reflection of motivation.

**Table 5:** CR and AVE values for second-order latent variables.

**Second-order latent variable**	**First-order latent variable**	**Loading**	**CR**	**AVE**
Capability	KS	0.849	0.907	0.765
	DM	0.888		
	BR	0.886		
Motivation	OP	0.659	0.867	0.482
	SR	0.686		
	EM	0.71		
	REI	0.722		
	INT	0.68		
	BCO	0.709		
	BCA	0.691		
Opportunity	VAL	0.765	0.882	0.600
	LS	0.732		
	PN	0.811		
	PE	0.816		
	EI	0.74		

**Figure 3:**
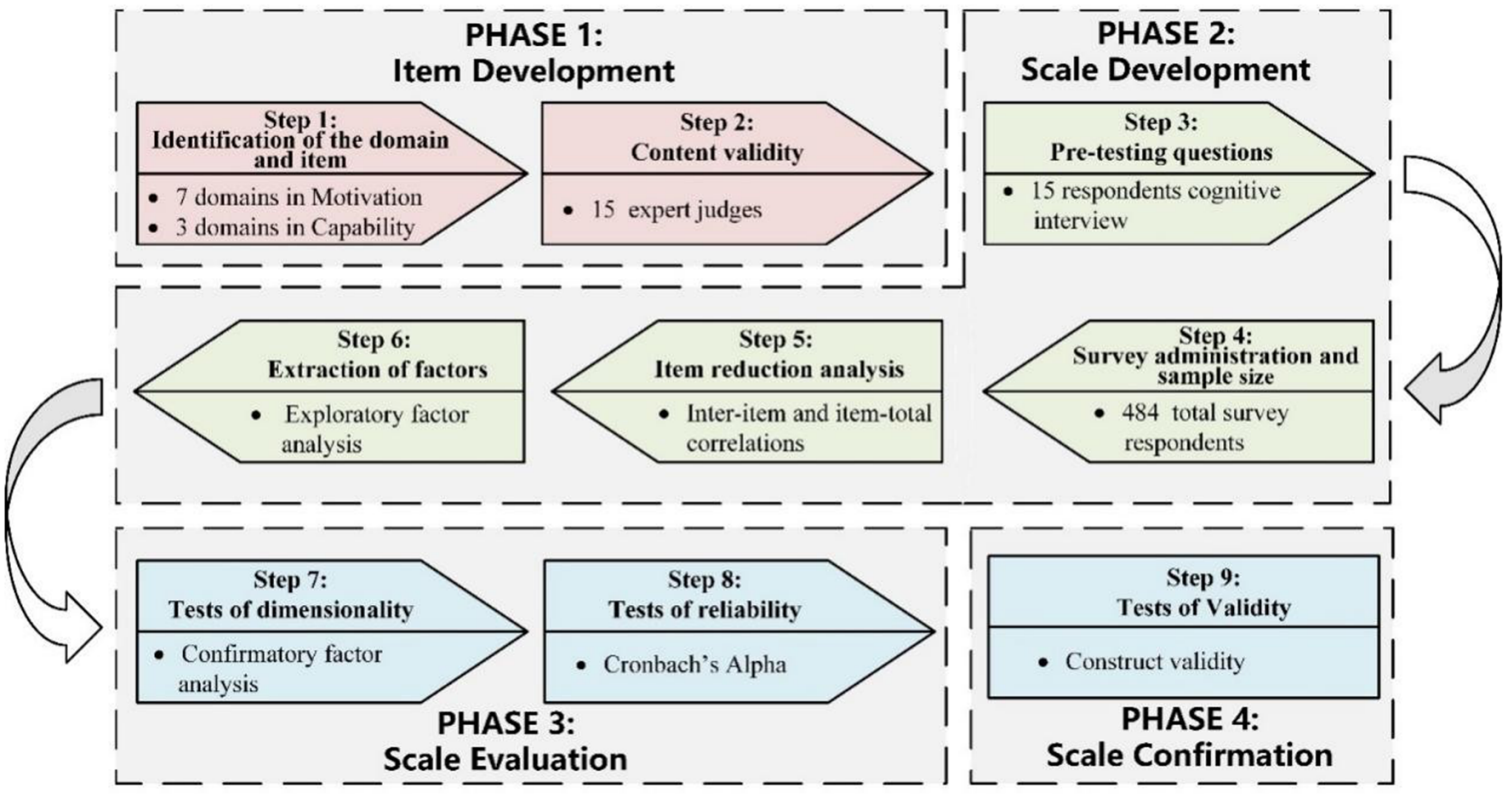
Confirmation factor analysis and path coefficients of the COM-B model.

The validation results of the structural equation model designed according to the COM-B model framework are shown in Figure 3. Capability, as a second-order latent variable, had little direct effect on Behavior (β = −0.04, *P* = 0.378) and was not statistically significant. Therefore, hypothesis 1 was not valid. The direct effect of Opportunity as a second-order latent variable on Behavior was positive and statistically reliable (β= 0.26, *p* < 0.001), and hypothesis 2 was supported. Motivation had the most significant direct positive effect on Behavior (β= 0.41, *p* < 0.001), as the strongest indicator in the COM construct, and thus hypothesis 3 was also supported. Within the inner COM model, the direct positive effect of both Capability and Opportunity on motivation was 0.21 (*p* < 0.001) and 0.18 (*p* < 0.001), respectively. For the test of mediating effects of Motivation (hypotheses 4 and 5), *n* = 5,000 bootstrap replicate sampling results can be inferred by bias-corrected 95% CI and percentile 95% CI results (Table 6). It was statistically significant that Motivation was a mediator of both the paths for Capability to Behavior and Opportunity to Behavior, respectively. Notably, Motivation fully mediated the relationship between Capability and Behavior, as the direct effect of Capability on Behavior was not statistically significant. In addition, Motivation served as a partial mediator in the pathway from Opportunity to Behavior. These findings support Hypotheses 4 and 5.

**Table 6:** Mediating effect test of motivation.

**Hypothesized paths**	**Effect type**	**Effect value**	**Standard error**	**Bias-corrected 95% CI**	***P* value**	**Percenntile 95% CI**	***P* value**
Capability → motivation → behavior	Direct effect	−0.042	0.039	(-0.128, 0.046)	0.357	(-0.129, 0.045)	0.35
	Indirect effect	0.086	0.026	(0.038, 0.142)	0.001	(0.036, 0.14)	0.001
Opportunity → motivation → behavior	Direct effect	0.26	0.046	(0.164, 0.346)	< 0.001	(0.166, 0.347)	< 0.001
	Indirect effect	0.074	0.023	(0.033, 0.123)	< 0.001	(0.03, 0.121)	0.001

### 4.3 Association rules from data mining

**Figure 4:**
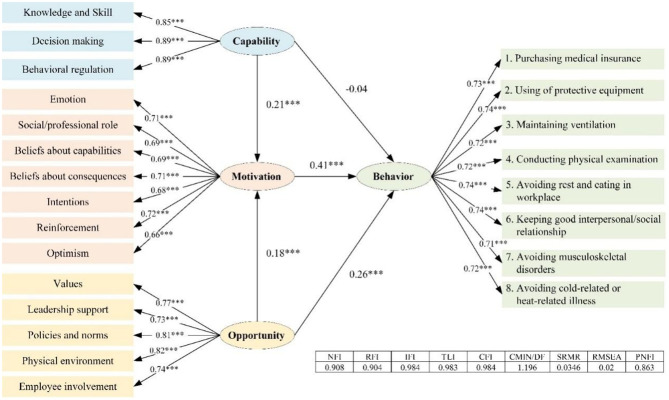
Support and Confidence distribution of association rule analysis results for behavior. ^***^P < 0.001.

This study positioned ARM as an exploratory data description tool for identifying co-occurrence patterns among variables, rather than for causal inference or hypothesis testing. A preprocessed dataset was conducted using Apriori ARM, according to the set parameters (minimum Confidence was 50%, minimum Support was 5%, Lift > 2) (14). A total of 123,324 association rule results were obtained by this set of parameters. These rules are categorized into eight specific CBOHR into “BEH-1 = H (High)” to “BEH-8 = H” and “BEH-1 = L(Low)” to “BEH-8 = Low”, for a total of 16 partitions. Items containing M (Middle) in each behavior partition were eliminated because we expected to get high-scoring domains associated with high-scoring behaviors and low-scoring domains associated with low-scoring behaviors. Middle-scoring domains and behaviors were excluded since the effect of these on behavior was insignificant. For BEH-2 = L, BEH-3 = L, and BEH-6 = L, we retained the domain with the highest score (M) because these domains had too few rules (13, 7, and 9 rules, respectively). Furthermore, for the domain of values inside Opportunities, the scores were only M and H, and therefore, we regarded M as L in this domain. After eliminating the middle-scoring domains, the final 3,028 rules remained. Their Support and Confidence distributions were shown in Figure 4. In each behavioral partition, the Support was mostly distributed in the interval of 5%−10%, and the Confidence distribution was concentrated in the interval of 50%−70%. The rules within the high-scoring behavioral partition were well above those within the low-scoring ones.

Next, we performed further rule extraction based on the Lift. Lift, an essential indicator in association rules, represents the concurrence frequency and relevance of domains (shown in Equation 3). According to Lift, the rules distributed in the 16 behavioral partitions were ranked, and we defined the top ten of Lift in each partition as strongly associated rules.

**Figure 5:**
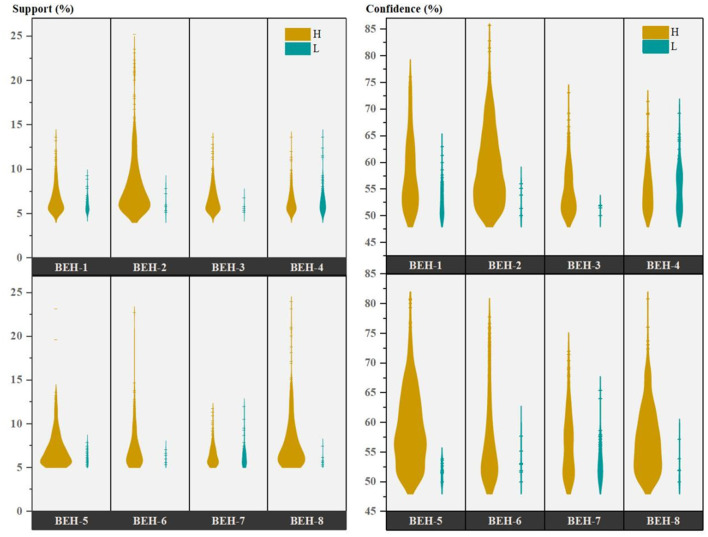
Strong association rule filtered from behavior = high.

Figure 5 showed the strong association domains for each behavioral partition when the BEH = H were available. In partitions with BEH = H, the strongly associated sets had at least three domains. Interestingly, the set of strongly correlated domains for each partition with BEH = H almost always contains VAL (Values) and PN (Policies and norms), which came from the Opportunity. In Motivation, the occurrence in the strongly associated sets varied in different Behavior partitions, and Supplementary Table S6 showed the frequency distribution of domains in the strong associated rules with BEH = High. For high-scoring behavioral partitions, the strongly correlated domains in Opportunity were PN and VAL. In addition, the strongly correlated domains from Motivation had significant differences. For example, for BEH-1=H, the strongly correlated domains in the Motivation were SR, REI, OP, and INT, while those for BEH-2=H were SR, BCO, and EM (domains appeared at least five times in 10 strong correlation rules). This can, at the same time, explain to some extent part of the reason for the low AVE of Motivation in the confirmation factor analysis of the COM-B model, which, of course, cannot be perceived as statistical validation. Also, the extent to which different domains in Motivation played an influential and mediating role may be at different levels for each CBOHR.

**Figure 6:**
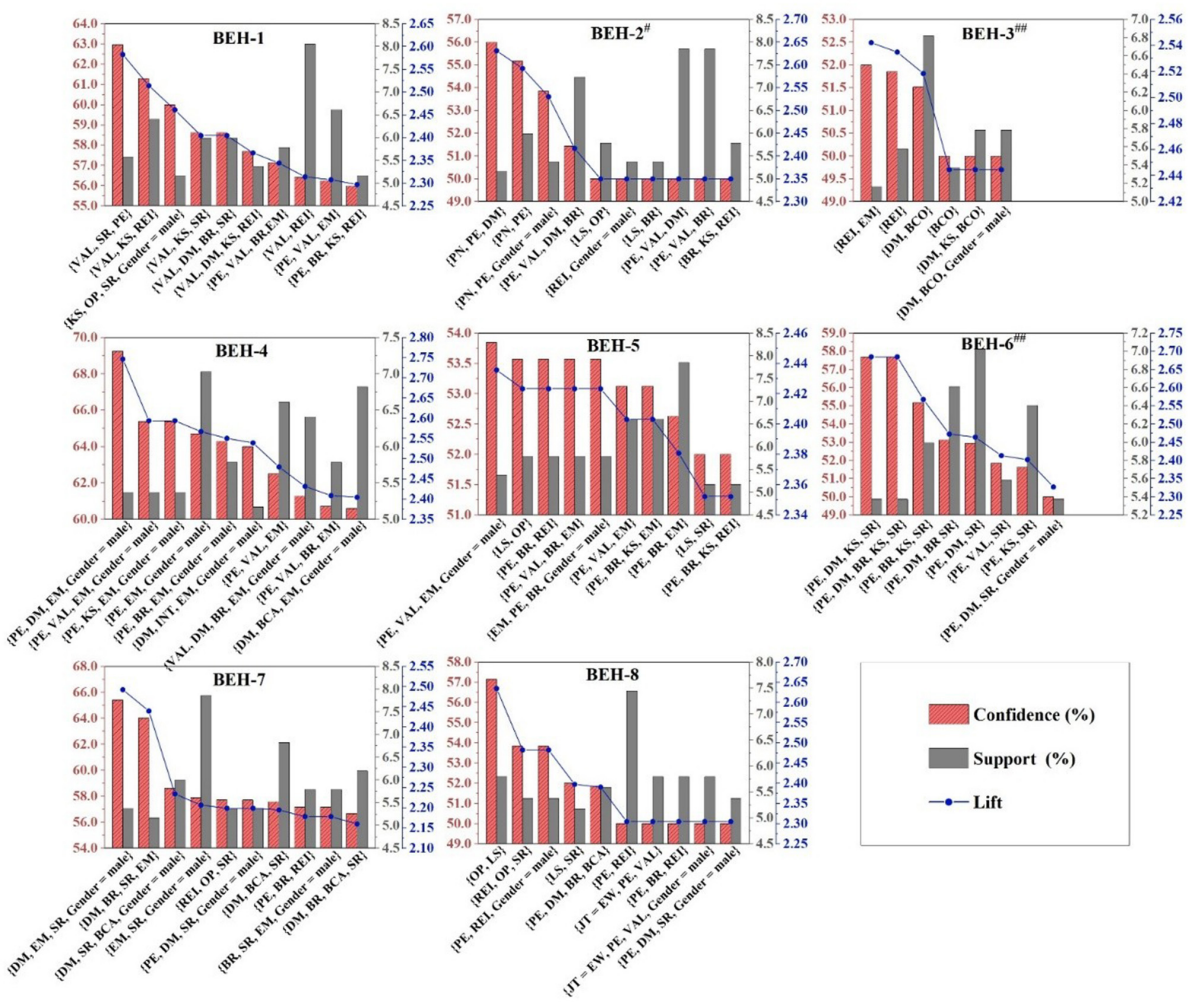
Strong association rule filtered from behavior = low. For BEH-2=L, BEH-3=L, and BEH-6=L, we retained the domain with its score was M because there were too few rules inside these domains, which were 13, 7, 9 rules respectively.

For low-scoring behaviors, the strong association domains for each behavioral partition exhibited distinct association characteristics compared to high-scoring ones (Figure 6). Supplementary Table S7 presents the frequency distribution of domains in strong association rules about BEH=L. The domains in Capability demonstrated a strong association with l BEH = L, which was not found in the strong association rules for BEH = H. It illustrated that low levels of CBOHR have a strong correlation with respondents' low Capability level. The sets appearing in Opportunity, other than VAL and PN, have PE as another frequent domain, indicating that BEH = L had a strong correlation with PE = L. The domains in Motivation appeared significantly less frequently than those where the behavior partition was high. This might be related to the fact that far fewer association rules that were above the required parameter threshold yielded in the low-scoring behavior partitions than in the high-scoring behavior partitions.

The analysis of these association results can inform interventions aimed at changing CBOHR. Traditional structural equation modeling (SEM) has been effective in identifying and predicting the effects of dependent variables (behaviors) and explaining the variance accounted for by latent variables. Quantitative analysis within SEM can reveal the path coefficients between second-order latent variables (modules). However, evaluating the relationship between first-order latent variables (domains) and outcome variables becomes challenging, particularly in complex global models. ARM provides a complementary approach, combining semi-quantitative and semi-qualitative methods to enhance or support statistical interpretation. Moreover, ARM can help identify the determinants of target behavior change from a qualitative perspective, enabling more precise identification of crucial elements for behavior intervention.

## 5 Discussion

### 5.1 Mediating effect for motivation

#### 5.1.1 Mediating effect of capability on behavior

**Figure 7:**
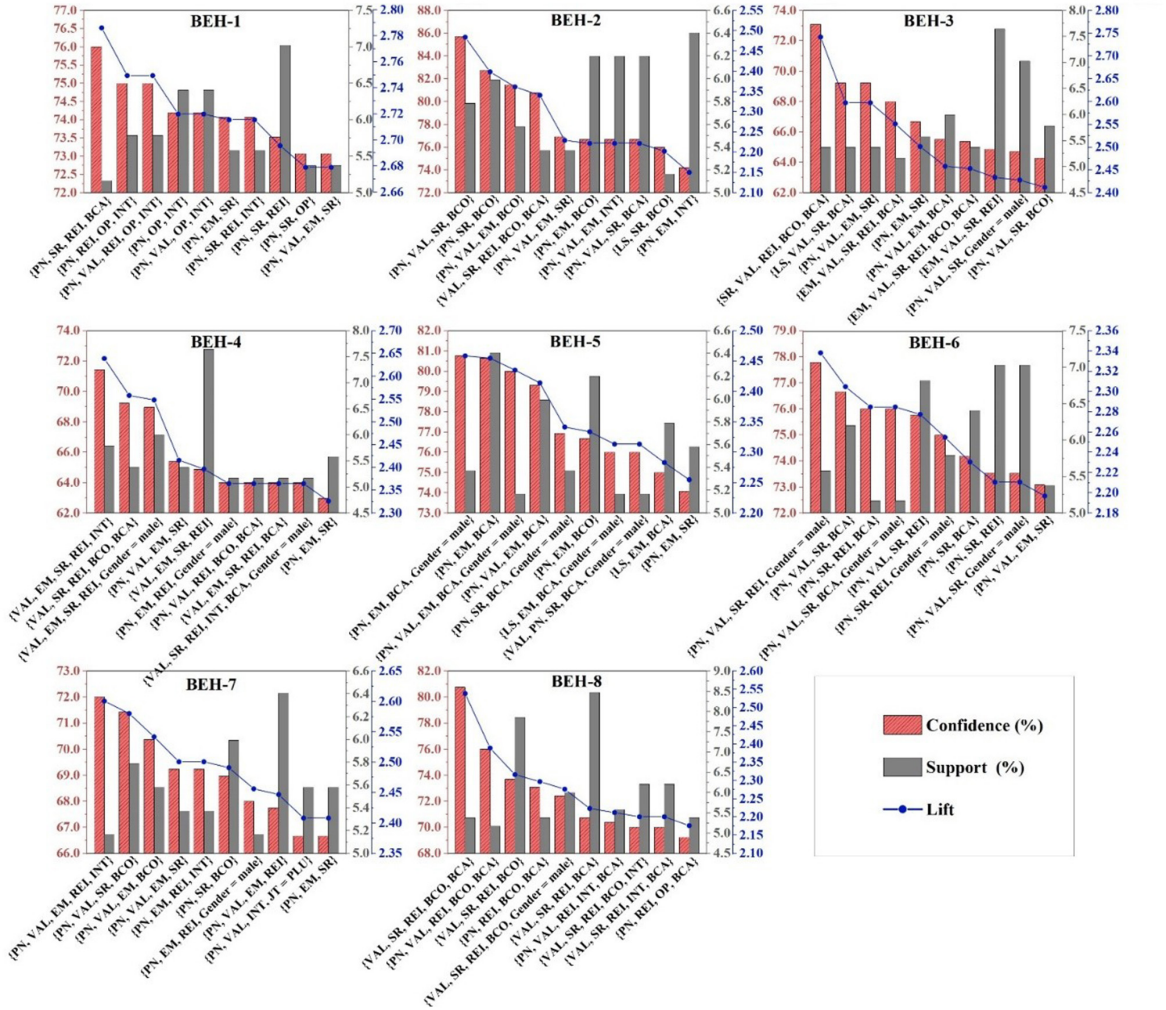
Individual chain evolution process for information processing of occupational health risk.

As shown in the results of the hypothesis test, there was no statistically significant direct effect from Capability to Behavior. Capability refers to the process of identifying and comprehending exposed occupational hazards through knowledge, skills, memory, and behavior management habits, all of which are encompassed within the Capability component. These elements are essential for managing occupational health risks in the workplace. Risk information is then processed during the cognitive risk phase, which involves both Capability and Motivation, as these factors directly influence the formation of risk-coping decisions. Therefore, we can assume that the individual chain evolution process for information processing of occupational health risk and coping implementation follows the following pattern: “identifying → cognizing → deciding → coping” (Figure 7).

In the sets of strongly correlated items where BEH=H (high behavior level), no domains from Capability appeared frequently. However, in the sets of strongly associated items, BEH, L, DM, KS, and BR appeared with varying degrees of frequency. For example, in the CBOHR partition related to “purchasing health insurance”(BEH-1), “KS = L” was strongly correlated with low-scoring behavior, suggesting that limited knowledge and skills (KS) may serve as a barrier to BEH-1. Within the Motivation category, SR and REI showed the strongest associations with BEH-1. For insurance-related activities and healthcare services, it is important to educate workers and provide clear guidance on how to maximize available healthcare resources at minimal cost. Interventions targeting OP and INT should be effectively combined with enhancements to KS, such as through clear communication about the availability and importance of health insurance services. Ensuring that workers are aware of the benefits—and have access to and confidence in using them—is essential for improving uptake and behavioral outcomes.

#### 5.1.2 Mediating effect of opportunity to behavior

The main concepts of Opportunity were social influence and environmental resources. Opportunity may not be involved at the beginning of the individual chain evolution process for information processing of occupational health risk and coping implementation, because there was no relevant evidence for the relationship between Opportunity and Capability in this research. The results of the structural equation modeling research, however, demonstrated that Opportunity may affect cognition, decision, and coping at the same time (Figure 7). In the health promotion (health protective behavior enhancement) model framework, interpersonal (social) influences as well as environmental influences were parallel with specific behavioral cognitions (Motivation), on the premise that personal factors were being considered at the forefront (114). Among the results of the association analysis, the strong association sets for BEH = H, PN, and VAL were the most remarkable Opportunity-based domains. They served as the strongest association domains of excellent CBOHR performance, but the engagement domains from Motivation were different for different types of behaviors. As a result, fostering positive values among construction workers was crucial to the success of CBOHR, and it was important to communicate the health concept of companies to employees. Moreover, PN was a key factor in promoting workers' CBOHR, especially for behaviors like the usage of protective equipment (BEH-2). BCO appeared in high frequency in the sets of strongly correlated partitions for BEH-2 = H and BEH-2 = L, indicating that the belief about consequence was critical in Motivation. To overcome the barriers to the usage of protective equipment by construction workers, it was important to reinforce their clear understanding of the effects of the equipment and let them know that adopting these measures can help them prevent negative health outcomes.

### 5.2 The significance of this research

#### 5.2.1 Contribution of the data results to objectives

Programs that promote worker health require sustained investment and long-term commitment. From the perspective of benefits, employers often do not have a positive attitude toward such initiatives, as substantial efforts cannot be immediately rewarded, and minimal efforts cannot be penalized (115). From the point of view of responsibility, factors such as high employee mobility and the cumulative nature of health damage over time make it difficult to hold employers accountable for work-related ill health, particularly in the construction sector, where workers frequently change employers. This reduces financial incentive for employers to invest in preventive health measures (116). From the viewpoint of performance, there were many determinants of worker health promotion, in addition to the fact that the desired effects may not occur in the short term and a lack of methods to make a measurable link between beneficial measures and performance (117). From the perspective of ownership, there were almost no medical personnel dedicated to managing workers' health on construction sites, and the involvement of managers was limited to superficial approaches. Staff felt it was more appropriate to hand over health management to health professionals (115).

Failure of adequate emphasis on occupational health management or epidemiological information management was seen as a barrier to OHS success (118). Therefore, an excellent OHS management system involves the adoption of preventive measures to reduce work-related risks by proactive action to improve workers' health, safety, and satisfaction. In China, it is extremely clear that the process for managing occupational health has gaps and that managers lack occupational health knowledge. Additionally, it is believed that the solution to this problem lies in the necessity for employees to develop a personal sense of accountability for health-related concerns. Especially at this stage of development, as seen in China, it is crucial to strengthen employees‘ own occupational health management to achieve maximum health risk reduction with the least amount of financial expenditure. Of course, employers and the government must continue to strengthen their functions and responsibilities while also improving the level of occupational health self-management among workers as a supporting role. This research can help behavior change practitioners understand the domains in which to implement interventions and build continuous intervention programs when they are aware that workers are lacking in a certain CBOHR habit. Furthermore, the research can provide evidentiary support for targeted intervention domains that correlate to distinct levels of behavior because overcoming behavioral difficulties is a sustained and phased process.

#### 5.2.2 Innovativeness of the method

Quantitative research by questionnaire can efficiently measure barriers and facilitators to the presence of CBOHR in construction workers and design applicable intervention programs through the theoretical domain framework of behavior change. Adopting a psychometric paradigm based on psychophysical scaling and multivariate analysis techniques can produce representations of individuals‘ internal attitudes on the effects of target behaviors. This approach is more likely to be adopted by managers and supervisors responsible for improving workers' health behavior on construction projects.

SEM can systematically identify facilitators and barriers; however, the results did not clearly highlight the most influential indicators within the COM constructs. In complicated models involving numerous variables, conventional statistical methods, such as correlation analysis, linear regression analysis, and SEM, may produce unstable or ambiguous conclusions, as they operate within a “hypothesis-testing” paradigm focused on confirming predefined effects among modules. As a result, findings from these statistical methods may not fully capture the intricacies of psychological and organizational management challenges. Data mining techniques, which are well-suited to analyzing large datasets with numerous variables, might provide a potential solution. By qualitatively processing questionnaire data, ARM could infer relationships between factors that are difficult to quantify or impossible to describe. This study innovatively combines the commonly used SEM with ARM techniques, which proved more effective in identifying key factors and mechanisms underlying CBOHR. The combination of quantitative and qualitative analyses may be more effective in assisting managers, practitioners, and researchers in designing CBOHR intervention plans that are scientifically grounded and more responsive to workers' evolving needs.

### 5.3 Limitation

There is currently no applicable scale for assessing the Capability, Motivation, and Behavior modules in the context of construction worker health. Therefore, we followed a conventional method by splitting the sample data into two parts for exploratory and confirmatory factor analyses, respectively. Measurement bias might exist in preliminary scales that had not undergone multi-case or large-sample validation. However, the scale's measuring function was partially supported by validity assessments conducted by occupational safety and health experts in the construction field. In addition, a sizable number of construction workers and related occupational health managers in the field participated in this research. The satisfactory reliability and validity results further support the scale's performance. The next step involves undertaking a larger range of research to confirm the scale's generalizability. This will also improve the questionnaire, equipping decision-makers in the construction industry with the necessary resources to gather more comprehensive research evidence for intervention adoption. The goal of this research was to provide decision-makers in occupational health improvement in the construction industry with the tools that obtain more comprehensive research evidence on intervention implementation.

This study did not validate the interaction between Capability and Opportunity. Similar to most studies that apply the unified framework of Capability, Opportunity, and Motivation, we treated the domains of Capability and Opportunity as parallel initial variables. Personal factors such as knowledge, memory, and experience can be transmitted to each other through interpersonal influences, which may exist within the group. However, one of our previous studies showed that, just like Chinese occupational groups such as miners and construction workers with high mobility, interpersonal influences among colleagues were not demonstrated. This did not confirm whether other domains of Opportunity (or culture) had an impact on the individual's ability. Therefore, this unexplored area may be worth exploring.

Occupational health risk factors regarding construction workers are numerous and may overlap with safety risk factors, making coping behaviors sometimes ambiguous or sometimes singular. The eight behaviors we screened may not provide complete coverage of construction workers' CBOHR in the workplace and thus are not considered representative. The purpose of this study was to investigate the mechanisms influencing CBOHR change in construction workers using the COM-B model combined with ARM. Considering the types of projects that may be necessary for subsequent project-based CBOHR studies, this paper aims to provide both theoretical and evidential support for intervention implementation.

## 6 Conclusion

This study empirically validates the COM-B framework within the occupational health context of Chinese construction workers for the first time. These findings extend the generalizability of COM-B beyond clinical and lifestyle settings, establishing a psychometrically robust instrument for assessing construction-specific capability, opportunity, and motivation.

The integration of COM-B with ARMRM demonstrates a transferable workflow: practitioners can rapidly identify culture-specific levers (values and policy norms) and design low-resource interventions without the need for longitudinal data. This proof-of-concept positions the framework as a ready-to-use toolkit for industry stakeholders seeking to reduce the occupational disease burden across diverse construction settings.

## Data Availability

The datasets presented in this study can be found in online repositories. The names of the repository/repositories and accession number(s) can be found in the article/Supplementary material.
