# Supplementary material for: Coping behavior toward occupational health risks among construction workers: determinant identification using the COM-B model and data mining analysis
Source: Front Public Health. 2025 Sep 12;13:1643332. doi: 10.3389/fpubh.2025.1643332 (PMC12463945; doi:10.3389/fpubh.2025.1643332)
Supplement: Supplementary file 1 [file Table_1.docx]

Table S1. Mapping of COM constructs to TDF domains and definitions of domains

| COM | TDF domain | Definition |
| --- | --- | --- |
| Capability | Knowledge | An awareness of the existence of something. |
|  | Skills | An ability or proficiency acquired through practice. |
|  | Memory, attention and decision processes | The ability to retain information, focus selectively on aspects of the environment and choose  between two or more alternatives. |
|  | Behavioural regulation | Anything aimed at managing or changing objectively observed or measured actions. |
| Opportunity | Environmental context and resources | Any circumstance of a person’s situation or environment that discourages or encourages the  development of skills and abilities, independence, social competence and adaptive behaviour. |
|  | Social influences | Those interpersonal processes that can cause individuals to change their thoughts, feelings,  or behaviours. |
| Motivation | Social/professional role and identity | A coherent set of behaviours and displayed personal qualities of an individual in a social or  work setting. |
|  | Beliefs about capabilities | Acceptance of the truth, reality or validity about an ability, talent or facility that a person can put  to constructive use. |
|  | Optimism | The confidence that things will happen for the best or that desired goals will be attained. |
|  | Beliefs about Consequences | Acceptance of the truth, reality, or validity about outcomes of a behaviour in a given situation. |
|  | Reinforcement | Increasing the probability of a response by arranging a dependent relationship, or contingency, between the response and a given stimulus. |
|  | Intentions | A conscious decision to perform a behaviour or a resolve to act in a certain way. |
|  | Goals | Mental representations of outcomes or end states that an individual wants to achieve. |
|  | Emotion | A complex reaction pattern, involving experiential, behavioural, and physiological elements, by which the individual attempts to deal with a personally significant matter or event. |

Table S2. The content and code of items in COM-B model

| **Code** | **Items**  (1 – strongly disagree, 5 - strongly agree) |
| --- | --- |
| **Capability** |  |
| Knowledge & Skills |  |
| KS1 | I can use some equipment and resources to (insert CBOHR). |
| KS2 | I am clear about when and how to implement (insert CBOHR) in my work. |
| KS3* | I know how to (insert CBOHR) in the work. |
| KS4 | I know that (insert CBOHR) can reduce my health damage caused by hazards at work. |
| Decision making |  |
| DM1* | I know taking different (insert CBOHR) to cope different health risks. |
| DM2 | I will pay attention to the health effects brought by environment when changing new workplace. |
| DM3 | I can pay attention to the change of body sensation during the work. |
| DM4 | I will not forget (insert CBOHR) in the work time. |
| Behavioural Regulation |  |
| BR1 | I will correct it immediately when I find that I am not doing a good job on the (insert CBOHR). |
| BR2 | I will continue to maintain or strengthen (insert CBOHR). |
| BR3* | I will do my best to create resources to (insert CBOHR) even if the organization's health resources are limited. |
| BR4 | I will learn knowledge and skills for health protection at work. |
| **Motivation** |  |
| Social/professional role |  |
| SR1 | I should be responsible for the implementation of (insert CBOHR) in my work. |
| SR2 | I implement (insert CBOHR) need extra support. |
| SR3 | My occupational environment requires that I must implement (insert CBOHR). |
| SR4* | I can keep up with the heavy workload while implementing (insert CBOHR). |
| Optimism |  |
| OP1 | Implementation of (insert CBOHR) can benefit me in the long time. |
| OP2* | Implementation (insert CBOHR) is a very simple thing. |
| OP3 | I can avoid many health issues in the future by implementing (insert CBOHR). |
| OP4 | Implementation (insert CBOHR) enables me to stay healthy at work. |
| Beliefs about consequences |  |
| BCO1 | It will make me trigger many diseases if failing to implement (insert CBOHR). |
| BCO2 | The physical illness brought by failure of implementing (insert CBOHR) will cause me great pain later in life. |
| BCO3* | The physical illness brought by failure of implementing (insert CBOHR) will drag my family down later. |
| BCO4 | The health issues brought by failure of implementing (insert CBOHR) may cause me to retire early. |
| Beliefs about capabilities |  |
| BCA1 | There is almost no difficulty can prevent me from implementing (insert CBOHR). |
| BCA2* | I have the ability to implement (insert CBOHR) even if the conditions of work environment are constrained. |
| BCA3 | I can consistently implement (insert CBOHR) in my work. |
| BCA4 | I can feel that my implementation of (insert CBOHR) has a tangible effect on my health. |
| Reinforcement |  |
| REI1 | The publicity of occupational disease prevention and control warns me paying attention to (insert CBOHR). |
| REI2 | The physical discomfort from work makes me pay attention to (insert CBOHR). |
| REI3* | It is intolerable for me about failure of (insert CBOHR) in the work. |
| REI4 | It makes me feel safe to implemented (insert CBOHR) at work. |
| Emotions |  |
| EM1 | I often feel anxious that I am not implementing (insert CBOHR) in my work. |
| EM2 | I am satisfied with the implementation of (insert CBOHR) in my work. |
| EM3* | I am not worried about my occupational health for the current health protection measures. |
| EM4 | I like to implement (insert CBOHR) because it brings us a healthy working environment. |
| Intentions |  |
| INT1 | Implementing (insert CBOHR) at work can make me avoid work-related illnesses. |
| INT2 | Implementing (insert CBOHR) at work can extend my working years and thereby earning more money. |
| INT3* | Implementing (insert CBOHR) is one of the tasks to be completed. |
| INT4 | Implementing (insert CBOHR) can make me more comfortable to work. |
| **Opportunity** |  |
| Leader support |  |
| LS1 | I believe company leaders are the main responsible for our occupational health. |
| LS2 | I believe leaders or companies value the health and wellness of their employees. |
| LS3 | My foreman will always remind me to check or change my protective equipment. |
| LS4 | My foreman pays close attention to personal protection on site. |
| LS5 | My foreman is very concerned about the contamination of hazards in the workplace environment. |
| Value |  |
| VAL1 | I think it is important to put your own safety and health first. |
| VAL2 | I believe that protecting occupational health is a way to increase the long-term profitability of a company. |
| VAL3 | I believe that work-related diseases can be prevented. |
| Physical environment |  |
| PE1 | I can perceive the existence of occupational hazards in the workplace environment. |
| PE2 | I feel that the protection system is effective in the workplace environment. |
| PE3 | I think it is reasonable to place equipment in the workplace. |
| PE4 | We regularly clean and inspect occupational health hazards. |
| Policy and norms |  |
| PN1 | I think that the medical examination set by the company for us is reasonable. |
| PN2 | I think that the company will transfer my position according to my health condition. |
| PN3 | I think that the company will advise us to purchase other commercial medical insurance. |
| PN4 | I know how to respond to occupational health emergencies. |
| PN5 | We will regularly check or replace protective equipment. |
| Employee involvement |  |
| EI1 | The company often trains us on how to prevent work-related diseases. |
| EI2 | The foreman usually informs us the occupational hazards in the workplace and discuss the solutions with us. |
| EI3 | The foreman will seriously consider our comments on the cleaner production program. |
| EI4 | Our work team will listen to our views when developing occupational health policies. |
| **Behaviour** | (1 – strongly inconsistent, 5 - strongly consistent) |
| BEH-1 | Purchasing medical insurance |
| BEH-2 | Using of protective equipment |
| BEH-3 | Maintaining ventilation |
| BEH-4 | Conducting physical examination |
| BEH-5 | Avoiding rest and eating in workplace |
| BEH-6 | Keeping good interpersonal/social relationship |
| BEH-7 | Avoiding musculoskeletal disorders |
| BEH-8 | Avoiding cold-related or heat-related illness |

Note: Items with * were deleted according to factor analysis.

Table S3 Demographic information of the respondents

| **Variable** | **Number**  (**Proportion, %**) | **Variable** | **Number**  **(Proportion, %)** |
| --- | --- | --- | --- |
| **Gender** |  | **Length of work** |  |
| Male | 409 (84.50) | < 5 years | 34 (7.02) |
| Female | 75 (15.50) | 6~10 years | 115 (23.76) |
| **Age** |  | 11~15 years | 284 (58.68) |
| 18~30 | 5 (1.03) | 16~20 years | 51 (10.54) |
| 31~40 | 59 (12.19) | > 20 years | — |
| 41~50 | 212 (43.80) | **Job type of construction workers** |  |
| 51~60 | 174 (35.95) | Polisher (POL) | 35 (7.23) |
| > 60 | 34 (7.02) | Electric welder (EW) | 38 (7.85) |
| **Education level** |  | Plumber (PLU) | 74 (15.29) |
| Primary school or below | - | Concrete worker (COW) | 56 (11.57) |
| Junior high school | 161 (33.26) | Equipment installer (EQI) | 35 (7.23) |
| High school or technical secondary school | 271 (55.99) | Scaffolder (SCA) | 33 (6.82) |
| Junior college | 49 (10.12) | Carpentry (CAR) | 34 (7.02) |
| Bachelor or above | 3 (0.61) | Bricklayer (BRI) | 49 (10.12) |
| **Month income** |  | Stoneworker (STO) | 10 (2.07) |
| < 5000 | 54 (11.16) | Decoration workers (DW) | 45 (9.30) |
| 5000~7000 | 20 (4.13) | Tower crane commander (TCC) | 7 (1.45) |
| 7000~9000 | 163 (33.68) | Tower crane driver (TCD) | 14 (2.89) |
| 9000~11000 | 158 (32.64) | Elevator operator (ELO) | 23 (4.75) |
| > 11000 | 89 (18.39) | Factotum (FAC) | 31 (6.40) |

The respondents we surveyed were frontline workers from construction site, and they were exposed to various serious occupational hazards. We surveyed 14 job types in construction site, and Table 1 showed the demographic information of the construction workers respondents. Due to working environment, male construction workers were the majority, accounting for 84.5%. About 79 percent respondents in this research were in the age group between 41 and 60, and most of them received secondary education (89%). For income level, 84.71 percent of the respondents earned more than 7,000 yuan per month. Moreover, nearly 89% of the respondents had less than 15 years of work.

Table S4. Rotated component matrix of capability scale

| 题项 | 成分 | | | |
| --- | --- | --- | --- | --- |
|  | 1 | 2 | 3 | 4 |
| BR-3 | **0.846** | 0.141 | 0.144 | 0.036 |
| BR-2 | **0.836** | 0.197 | 0.094 | 0.024 |
| BR-4 | **0.777** | 0.321 | 0.306 | 0.080 |
| BR-1 | **0.682** | 0.213 | 0.319 | -0.069 |
| KS-2 | 0.151 | **0.839** | 0.142 | -0.100 |
| KS-4 | 0.240 | **0.807** | 0.128 | 0.020 |
| KS-1 | 0.383 | **0.719** | 0.322 | 0.061 |
| DM-4 | 0.238 | 0.284 | **0.754** | 0.075 |
| DM-2 | 0.270 | 0.312 | **0.670** | 0.284 |
| DM-3 | 0.419 | 0.431 | **0.636** | 0.155 |
| KS-3 | 0.098 | -0.154 | 0.607 | -0.534 |
| DM-1 | 0.051 | -0.092 | 0.176 | 0.817 |

Table S5. Fit results of constructs of Capability, Motivation and Opportunity

| Models |  | χ^2^ | df | χ^2^ /df | GFI | AGFI | NFI | TLI | CFI | RMSEA | SRMR |
| --- | --- | --- | --- | --- | --- | --- | --- | --- | --- | --- | --- |
| Factor model of Capability |  |  |  |  |  |  |  |  |  |  |  |
|  | Pre-modification | 72.384 | 32.000 | 2.262 | 0.941 | 0.898 | 0.955 | 0.964 | 0.974 | 0.072 | 0.043 |
|  | post-modification | 45.143 | 24.000 | 1.881 | 0.958 | 0.922 | 0.968 | 0.977 | 0.985 | 0.060 | 0.037 |
| Factor model of Motivation |  |  |  |  |  |  |  |  |  |  |  |
|  | No modification | 201.534 | 168 | 1.2 | 0.929 | 0.903 | 0.941 | 0.987 | 0.989 | 0.029 | 0.0318 |
| Factor model of Opportunity |  |  |  |  |  |  |  |  |  |  |  |
|  | No modification | 543.236 | 179 | 3.035 | 0.897 | 0.867 | 0.94 | 0.951 | 0.959 | 0.065 | 0.0339 |

Table S6. Frequency distribution of domains in BEH = High strong association rule

| Antecedent | Consequent | Frequency | Antecedent | Consequent | Frequency |
| --- | --- | --- | --- | --- | --- |
| BEH1 = H | PN = H | 1 | BEH5 = H | PN = H | 0.8 |
|  | VAL = H | 0.3 |  | VAL = H | 0.3 |
|  | SR = H | 0.6 |  | EM = H | 0.8 |
|  | REI = H | 0.5 |  | BCA = H | 0.8 |
|  | OP = H | 0.5 |  | SR = H | 0.3 |
|  | INT = H | 0.5 |  | Gender = Male | 0.5 |
|  | EM = H | 0.2 | BEH6 = H | PN = H | 1 |
|  | BCA = H | 0.1 |  | VAL = H | 0.6 |
| BEH2 = H | PN = H | 0.8 |  | SR = H | 1 |
|  | VAL = H | 0.6 |  | EM = H | 1 |
|  | SR = H | 0.6 |  | REI = H | 0.5 |
|  | BCO = H | 0.6 |  | BCA = H | 0.4 |
|  | EM = H | 0.5 |  | Gender = Male | 0.4 |
|  | BCA = H | 0.2 | BEH7 = H | PN = H | 0.9 |
|  | INT = H | 0.2 |  | VAL = H | 0.6 |
|  | REI = H | 0.1 |  | EM = H | 0.7 |
| BEH3 = H | VAL = H | 0.9 |  | REI = H | 0.4 |
|  | PN = H | 0.5 |  | SR = H | 0.4 |
|  | SR = H | 0.9 |  | INT = H | 0.3 |
|  | BCA = H | 0.5 |  | BCO = H | 0.3 |
|  | EM = H | 0.5 |  | Gender = Male | 0.1 |
|  | BCO = H | 0.3 |  | JP = PLU | 0.1 |
|  | REI = H | 0.1 | BEH8 = H | VAL = H | 0.8 |
|  | LS = H | 0.1 |  | PN = H | 0.4 |
| BEH4 = H | VAL = H | 0.8 |  | REI = H | 1 |
|  | PN = H | 0.4 |  | BCA = H | 0.7 |
|  | SR = H | 0.8 |  | SR = H | 0.6 |
|  | REI = H | 0.8 |  | BCO = H | 0.6 |
|  | EM = H | 0.7 |  | INT = H | 0.3 |
|  | BCA = H | 0.4 |  | OP = H | 0.1 |
|  | INT = H | 0.2 |  | Gender = Male | 0.1 |
|  | BCO = H | 0.2 |  |  |  |

Table S7. Frequency distribution of domains in BEH = Low strong association rule

| Antecedent | Consequent | Frequency | Antecedent | Consequent | Frequency |
| --- | --- | --- | --- | --- | --- |
| BEH1 = L | VAL = M | 0.8 | BEH5 = L | PE = L | 0.8 |
|  | PE = L | 0.4 |  | VAL = M | 0.3 |
|  | KS = L | 0.5 |  | BR = L | 0.6 |
|  | BR = L | 0.3 |  | LS = L | 0.2 |
|  | DM = L | 0.2 |  | KS = L | 0.2 |
|  | SR = L | 0.4 |  | EM = L | 0.6 |
|  | REI = L | 0.4 |  | Gender = Male | 0.2 |
|  | EM = L | 0.2 |  | OP = L | 0.2 |
|  | OP = L | 0.1 |  | REI = L | 0.2 |
|  | Gender = Male | 0.1 | BEH6 = L## | PE = L | 1 |
| BEH2 = L # | PN = L | 0.5 |  | VAL = M | 0.1 |
|  | PE = L | 0.5 |  | DM = L | 0.6 |
|  | VAL = M | 0.3 |  | KS = L | 0.4 |
|  | EI = M | 0.2 |  | BR = L | 0.2 |
|  | LS = L | 0.1 |  | SR = L | 1 |
|  | BR = L | 0.4 |  | Gender = Male | 1 |
|  | DM = L | 0.3 | BEH7 = L | PE = L | 0.2 |
|  | BCO = M | 0.7 |  | DM = L | 0.6 |
|  | REI = L | 0.3 |  | BR = L | 0.4 |
|  | OP = L | 0.1 |  | SR = L | 0.9 |
|  | Gender = Male | 0.2 |  | Gender = Male | 0.6 |
| BEH3 = L## | PE = M | 0.3 |  | EM = L | 0.4 |
|  | EI = M | 0.1 |  | BCA = L | 0.3 |
|  | LS = M | 0.1 |  | REI = L | 0.2 |
|  | DM = L | 0.4 |  | OP = L | 0.1 |
|  | KS = L | 0.1 | BEH8 = L | PE = L | 0.7 |
|  | BCO = L | 0.6 |  | LS = L | 0.2 |
|  | INT = M | 0.6 |  | VAL = M | 0.2 |
|  | REI = L | 0.3 |  | DM = L | 0.2 |
|  | EM = L | 0.1 |  | BR = L | 0.2 |
| BEH4 = L | PE = L | 0.7 |  | REI = L | 0.4 |
|  | VAL = M | 0.4 |  | SR = L | 0.3 |
|  | DM = L | 0.4 |  | OP = L | 0.2 |
|  | BR = L | 0.3 |  | BCA = L | 0.1 |
|  | KS = L | 0.1 |  | Gender = Male | 0.3 |
|  | EM = L | 1 |  | JT = EW | 0.2 |
|  | INT = L | 0.1 |  |  |  |
|  | BCA = L | 0.1 |  |  |  |
|  | Gender = Male | 0.8 |  |  |  |
